# The quality of Indigenous identification in administrative health data in Australia: insights from studies using data linkage

**DOI:** 10.1186/1472-6947-12-133

**Published:** 2012-11-16

**Authors:** Sandra C Thompson, John A Woods, Judith M Katzenellenbogen

**Affiliations:** 1 Combined Universities Centre for Rural Health, University of Western Australia, PO Box 109, Geraldton, WA, 6530, Australia; 2 Curtin Health Innovation Research Institute, Curtin University, GPO Box U1987, Perth, 6845, Australia

## Abstract

**Background:**

Missing or incorrect Indigenous status in health records hinders monitoring of Indigenous health indicators. Linkage of administrative data has been used to improve the ascertainment of Indigenous status. Data linkage was pioneered in Western Australia (WA) and is now being used in other Australian states. This systematic review appraises peer-reviewed Australian studies that used data linkage to elucidate the impact of under-ascertainment of Indigenous status on health indicators.

**Methods:**

A PubMed search identified eligible studies that used Australian linked data to interrogate Indigenous identification using more than one identifier and interrogated the impact of the different identifiers on estimation of Indigenous health indicators.

**Results:**

Eight papers were included, five from WA and three from New South Wales (NSW). The WA papers included a self-identified Indigenous community cohort and showed improved identification in hospital separation data after 2000. In CVD hospitalised patients (2000–05), under-identification was greater in urban residents, older people and socially more advantaged Indigenous people, with varying algorithms giving different estimates of under-count. Age-standardised myocardial infarction incidence rates (2000–2004) increased by about 10%-15% with improved identification. Under-ascertainment of Indigenous identification overestimated secular improvements in life expectancy and mortality whereas correcting infectious disease notifications resulted in lower Indigenous/ non-Indigenous rate ratios. NSW has a history of poor Indigenous identification in administrative data systems, but the NSW papers confirmed the usefulness of data linkage for improving Indigenous identification and the potential for very different estimates of Indigenous disease indicators depending upon the algorithm used for identification.

**Conclusions:**

Under-identification of Indigenous status must be addressed in health analyses concerning Indigenous health differentials – they cannot be ignored or wished away. This problem can be substantially diminished through data linkage. Under-identification of Indigenous status impacts differently in different disease contexts, generally resulting in under-estimation of absolute and relative Indigenous health indicators, but may perversely overestimate Indigenous rates and differentials in the setting of stigma-associated conditions such as sexually-transmitted and blood-borne virus infections. Under-numeration in Census surveys also needs consideration to address the added problem of denominator undercounts.

## Background

### Importance of health data for monitoring the health of the population and vulnerable groups

Health-related epidemiological and statistical information provides the basis for evidence and health policy. Accurate and reliable data are critical to monitoring the health of populations, particularly the health of minority and vulnerable populations. The importance and uses of quality health surveillance information are shown below, and include consideration of health in minority groups:

• Monitoring and surveillance of population health status, diseases, determinants of health, and health inequities between population subgroups

• Monitoring and surveillance of population health status, diseases, determinants of health, and health inequities between population subgroups

• Planning and developing policies, programs and services – at all levels, from National to local community

• Evaluating policies, programs and services

• Determining whether funding is adequate, distributed equitably, and used effectively and efficiently

• Facilitating administrative accountability

• Aiding advocacy efforts

• Raising community awareness

• Supporting high quality public health research

Without accurate data there is little capacity to monitor changes in health status, to evaluate access to services and the response of services to needs, or to quantify the resources expended on health services and programs. Arguably, without robust data, there is little accountability at political, policy and implementation levels.

There is great variation in the definition of Indigenous people in different international contexts [1]. Not only are definitions contested, the recording of Indigenous status in marginalised groups is complex and known to be poor in the administrative health data of many jurisdictions. Indigenous health information was recognised as an Australian health priority in the 1995 National Health Information Development Plan [2] which, after an Australia-wide consultation, led to the development of the National Aboriginal and Torres Strait Islander Health Information Plan [3].

Aboriginal and Torres Strait Islander (hereafter Indigenous) people are a unique group in Australia; they are the most marginalised of any identifiable group, featuring consistently at the lowest point on any marker of disadvantage [4-8]. Although individual markers of disadvantage are not unique to Indigenous Australians, for example, poverty and unemployment are by no means confined to Indigenous people, the coalescence of markers of disadvantage and the health outcomes that flow from them justify special attention. This was recognised by the Rudd government with its historic Apology to the Stolen Generations [9] and its commitment to closing the gap in life expectancy within a generation [10,11]. Questions which arise in these circumstances are how accurately the measurement of Aboriginal health disadvantage is measured, particularly given their heterogeneity with respect to language group and culture, and whether it is sufficiently sensitive to real changes in health and life expectancy. Fundamental to these questions is the ability to identify Indigenous people accurately and reliably in administrative health data. This is important not only in terms of health monitoring but also to facilitate access to health care entitlements targeting Indigenous people in order to improve Indigenous health outcomes [12].

### Identification of Indigenous Australians

There are three components to the definition of an Aboriginal and/or Torres Strait Islander Australian: descent, self-identification and community acceptance [13]. For most administrative data collections, self-identification only is used to collect information on Indigenous status. The Australian Bureau of Statistics uses a standard question on the Census form [8] and recommends that the same question is asked for Aboriginal and Torres Strait Islander identification in other contexts. Adherence to this recommendation varies substantially between individual Australian States, on account of their differing demographic profiles and their jurisdiction over hospitals in the federal political structure.

In terms of measuring improvements in the health status of Indigenous people, misclassification of identity can have a profound effect on health events and outcomes analyses. The quality of administrative data on Indigenous status varies between Australian jurisdictions, with the Northern Territory, which has the largest proportion of Indigenous residents (31.6%), considered to have the best ascertainment. Western Australia (WA), the largest, most sparsely populated state of Australia and home to 15% of Indigenous Australians (estimated 58,480 Indigenous people in 2006) [14] has been considered one of the jurisdictions with acceptable quality of Indigenous identification.

In WA, a state-wide hospital demographic validation survey was conducted by Young from July 2000 to January 2001. Based on 10,106 face-to-face patient interviews in 26 randomly selected government hospitals, it compared information collected directly from the individual with that on the patient’s hospital record [15]. Only 85.5% of the patients who identified as Aboriginal and Torres Strait Islander at interview were recorded as such in hospital records. The level of concordant identification was much higher in more remote regions (for example 93.5% in the Kimberley /Pilbara regions) and worst in the Perth metropolitan area (78.3%). Based upon these findings, Young developed different correction factors that could be applied to different health regions, with a correction factor for state-wide data of 1.09 [15]. The following year the Australian Institute of Health and Welfare (AIHW) adjusted this factor for WA to 1.06, without explicit justification but presumably due to re-weighting of results [16].

The AIHW in 2010 reported an overall improvement in the quality of Indigenous identification in hospital separations around Australia, albeit with considerable variation in quality between jurisdictions [17]. The results for the 966 patients who participated in WA suggest that Indigenous identification has improved in WA to the extent that 98% of Indigenous people and 99% of non-Indigenous people were correctly identified in the admission record. The identification for WA (where Indigenous people comprise 3.8% of the population) was thus considered to be almost as good as the best-performing jurisdiction, Northern Territory ( [14], with a weighted completeness of 97% (95%CI 95-99%) and a recommended correction factor of 1.03.

The Young and AIHW studies reported only the concordance between hospitalised patients’ responses to a specific interview question on Indigenous identification and their Indigenous status in the hospital record. However, there are additional approaches based on administrative sources of health data that can be used to explore Indigenous identification. The Western Australian Data Linkage System (WADLS) [18], which systematically links health data from core administrative databases and has been widely used for population health research, allows a way of examining the consistency of and (secular) trends in Indigenous identification reporting in different data sets. With data linkage systems currently being established in other Australian jurisdictions, it is timely to review studies investigating their utility as a means of improving Indigenous identification.

### The Western Australian Data Linkage System (WADLS)

**Figure 1:**
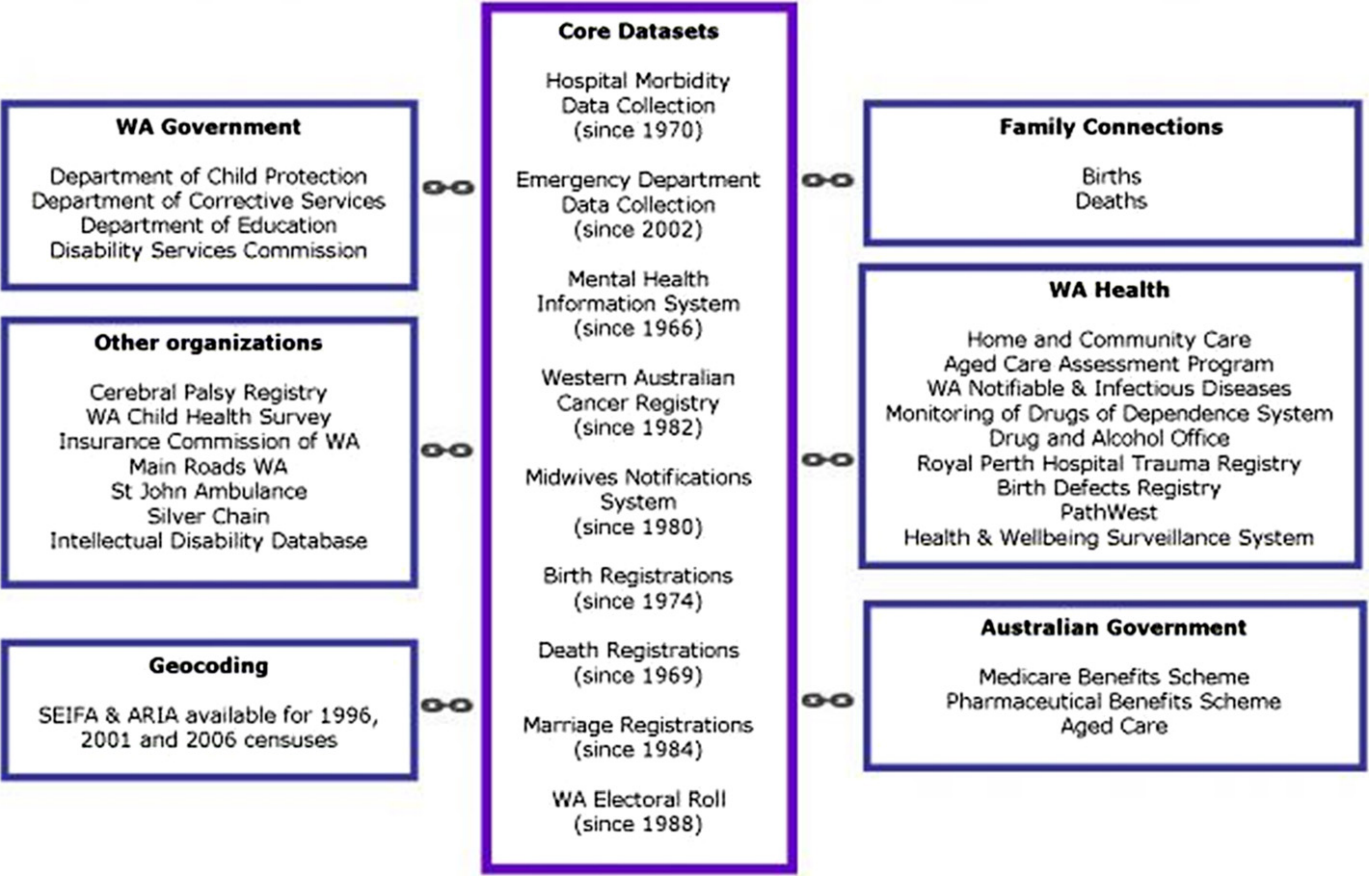
The WA Data Linkage System.

Western Australia (WA) is the state in Australia with the most extensive experience of using linked data, having established the first Australian data linkage system in 1995. The WADLS was a collaborative development by the University of Western Australia (UWA) and the WA Department of Health, and is one of a small number of comprehensive record linkage systems worldwide. Since 1995, a Data Linkage Unit based at the Department of Health has developed and maintained a system of linkages connecting health event data and other information relevant to health for individuals in WA. These linkages are created and maintained using rigorous, internationally accepted privacy-sensitive protocols, with probabilistic matching based on multiple data fields, name compression algorithms, multiple matching passes, and extensive clerical review of all potential linkages that are not identified as definite matches [19]. The WA Data Linkage System is not a data repository but instead provides pointers to original data sources maintained by separate data custodians. The core Data Linkage System consists of links within and between seven state-based core population health datasets, spanning up to 40 years (Table 1), augmented through further links to an extensive collection of additional state-based and nationwide administrative databases as well as research and clinical datasets (Table 1 and Figure 1).

**Table 1:** **Example of a data linkage system**: **core datasets of the WA data linkage system**

**Dataset**	**Information Collected (All include some demographic information)**	**Years of Data**	**Who Collects Indigenous identity information**
Hospital Morbidity Data Set (HMDS)	Patient separations from hospital – reasons for admission, illnesses, details of discharge etc.	1970 -	Hospital administrative staff (Admission clerks, ward clerks)
Mortality Data	Registrar General records information on all deaths in WA including date and cause of death	1969 -	Funeral directors, may be modified by ABS (see Figure 2)
Mental Health Database System (MHDS)	Patient separations from mental health facilities - reasons for admission, illnesses etc.	1966 -	Hospital administrative staff (Admission clerks, ward clerks)
WA Cancer Registry	Notification of incident cancers	1981 -	Diagnosing doctors and laboratories
Midwives Data Collection	Information on all births in WA, only the mother’s Indigenous identity is recorded in relation to the newborn	1980 -	Midwives
Birth Registry Data	Registrar General collects information on all births in WA including data and place of birth.	1974 -	Both parents are required to complete and sign the Birth Registration form
Electoral Roll	Every eligible Australian citizen (18 years or older) is required by law to enrol and vote		Not applicable (i.e. no information on Indigenous status is collected)

The WADLS is a powerful research tool, allowing the study of the entire WA population. An analysis of WADLS outputs from 1995–2003 estimated that 708 journal articles, reports, presentations, conference proceedings, theses and other items have been produced, reflecting the large research output from the WALDS. The ability to link information collected on the same individual in different settings and at different times across multiple datasets enables important and elegant public health research, including the evaluation of identification data. However, many of these outputs do not consider Australia’s Indigenous population, and those that include an analysis of Indigenous (health) indicators generally use only one Indigenous identifier, without further interrogation. For example, Hall and colleagues’ analysis of cancer outcomes in relation to Indigenous status stated “Due to variability in the recording of this status, any mention in any hospital, cancer or death record was added to the index admission record” [20]. Similarly, Moore et al. in their analysis of childhood acute lower respiratory tract infections commented “All datasets provided information concerning Aboriginal status and a child was identified as such if at least one record in one of the datasets recorded the child as Aboriginal” [21]. These analyses, while acknowledging the limitations of Indigenous identification, do not elucidate discordance between datasets or secular trends in the quality of administrative recording in this regard, or interrogate how shortcomings in Indigenous identification influence measurement of Indigenous health indicators. It is worth noting that without data linkage, epidemiological analyses based on administrative data are limited to using the Indigenous identifier pertaining to a single health event.

Concern over the shortcomings of Indigenous identification in administrative health datasets has prompted researchers in WA to undertake studies exploring Indigenous under-ascertainment in administrative health data using the opportunities provided by the WADLS. This paper systematically reviews published analyses in which person- or event-based Indigenous identification was interrogated as a means of improving its ascertainment. In recognition of the recent development of data linkage capacity in other Australian jurisdictions, our search was broadened to include eligible papers from these jurisdictions.

## Methods

**Figure 2:**
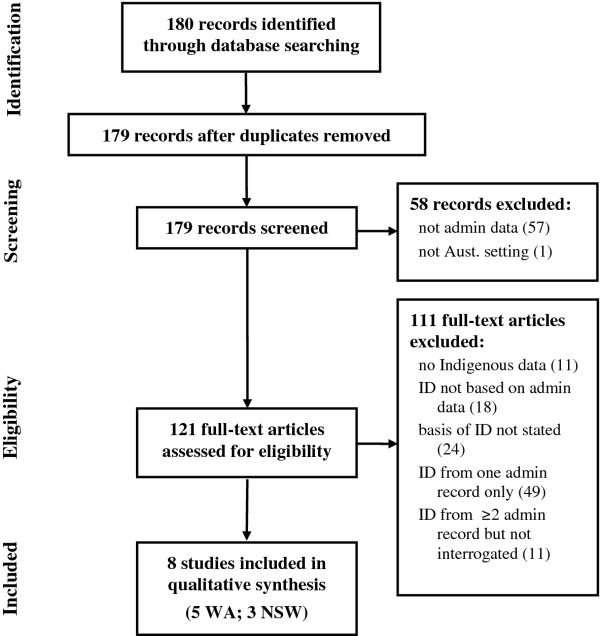
PRISMA flowchart showing process and findings related to article identification.

A search based on the PRISMA guidelines [22] was undertaken in PubMed for articles examining Indigenous/Aboriginal identification/status using data linkage in Australia. The search terms were (aborigin* OR indigenous) AND ((data OR record*) AND (linkage OR linked OR administrative)) AND (australia OR australia*) AND 1992:2012[dp]. After the first stage of searching and elimination of records not based on administrative data or pertaining to Australia, 121 full-text articles were assessed for eligibility. Studies considered eligible were those which incorporated explicit comparison of various strategies for Indigenous identification using data linkage, as a basis from which to evaluate or improve the overall identification of Indigenous status from administrative data. Most papers then interrogated the impact of the different identifiers on estimation of Indigenous health indicators. At least two authors reviewed the papers against the selected criteria, with any discrepancies resolved by discussion and consensus. The process and outcomes of the search as per the PRISMA guidelines are shown in Figure 2.

## Results

While there were many papers reporting analysis of linked data that reported Indigenous indicators, most were either silent on the exact nature of the data used for Indigenous status or reported the use of only one identifier for Indigenous identification. Typically, this was based on the identifier recorded during an index admission, or on the subject having been ever-identified as Indigenous in a record in any dataset. From 180 papers initially identified, after elimination of one unusual citation duplication in PubMed and records which did not specifically interrogate Indigenous status through the use of linked data, 8 studies were eligible for analysis (Table 2). Five of the published papers used the WADLS. Three papers used the more recently developed NSW Centre for Health Record Linkage (CHeReL).

**Table 2:** Summary of eight studies using Australian data linkage systems to examine Indigenous identification in administrative data

**1**^**st**^**Author**, **Year,State**	**Issue/Question**	**Study Approach**	**Findings**
Mak 2008 WA	• 26% of 7,619 notifications of STI/BBVs in WA in 2004 were missing information on Aboriginality.	Infectious disease notification data for STIs/BBVs from 2004, WADLS link to mortality, hospitalisation, midwives and mental health datasets. Utilised various definitions for Aboriginal	• Aboriginality able to be assigned for 74% of cases with a missing Aboriginal identifier
	• Can data linkage help with better estimation of the true rates of disease?	Sensitive – any Aboriginal identifier	• Determining Aboriginality via data linkage was significantly and independently associated with sex and disease
		Specific – identified as Aboriginal on notification form or consistently on data linkage	• Indigenous disease rates and the Indigenous /non-Indigenous rate ratios decreased with improved Indigenous identification
Bradshaw 2009 WA	• Has ascertainment of Indigenous status improved in hospital separation data over time?	1998/99 Aboriginal cohort, WADLS link, identification in hospital data in admissions 1980 to 2006	• Substantial variation in Indigenous coding
			• Improved Indigenous coding since 2002; sensitivity since 2002 of >90%
Draper 2009 WA	• Over the years 1997–2002, the proportion of deaths in WA increased steadily from 0.6% to 6.6%	Linked deaths of unknown Indigenous status in mortality records through WADLS to hospital, mental health and midwives data.	• Indigenous status assigned to most people.
	• Could data linkage be used to estimate missing Indigenous status and what were the effects on life expectancy and mortality rates?	M1 = most frequent count	• “Unknowns” proportionately more likely to be Indigenous
		M2 = any Indigenous identification	• Under-ascertainment leads to elevated life expectancy and lower mortality rates
Briffa 2010 WA	• What is the effect of different algorithms for identifying Indigenous status through data linkage?	Patients hospitalised with CVD, WADLS linked 20 yr history.	• Modest increases by linkage using 50% of admissions
	• What are the demographic factors most associated with under-ascertainment	1. Index admission (baseline comparator)	• 20.8% increase if identified on one admission or death record
		2. Index admission or subsequent death record flag	• Older, less disadvantaged and urban living more likely to be under-identified
		3. At least 50% of admissions recorded as Indigenous	
		4. Indigenous on at least 1 admission or death record5. Indigenous on at least 1 admission or death record	
Katzenellenbogen 2010 WA	• Individuals may have inconsistent coding of Indigenous status in hospital records and/or be coded different on mortality data	Index = Acute MI in hospital or death data	For Indigenous identified patients on inclusive definition
	• What is the impact of 2 different methods for ascertainment of Indigenous status on acute myocardial infarction and 28-day case fatality rates in Indigenous people?	WADLS - admissions since 1980 and death data	• age standardised rates and age-standardised rate ratios were higher
		Sensitivity analysis	• case fatality was reduced
		Inclusive = ever identified Indigenous	
		Restricted = coded as Indigenous on incident or death data	
Neville 2011 NSW	• Can reporting of deaths among Indigenous people in NSW on the ABS mortality be improved by record linkage with the NSW Admitted Patient Data Collection (APDC)?	ABS mortality data for 2002–2006 were linked with APDC. Six algorithms were developed	• Maximised enhancement occurred with “Indigenous ever” but Algorithms 5 and 6 were considered most methodologically sound.
	• To investigate specific sources of bias caused by record linkage	1. Baseline = reporting based on ABS mortality	• Algorithms 3–5 relatively similar enhancement and relatively unaffected by the number of years of APDCs linked.
		2. Ever reported as Indigenous	• Enhancement in identification:
		3. Proportional record criterion (>50%)	○ varied by age (most in children 5–9 years and those> 85 years)
		4. Proportional facility-level criterion or ABS mortality data	○ was higher in females
		5. Proportional records and proportional facility criteria (50% of record in 50% of facilities) or ABS mortality data	○ was greater in urban areas
		6. Two or more records in two or more facilities in the APDC or ABS mortality data	
		Also used different years of APDC and explored impact of age, sex and remoteness	
Xu 2011 NSW	• To improve the statistical ascertainment of Indigenous mothers in NSW by linking the Midwives Data Collection (MDC) (which records the Indigenous status of the mother only) and the Indigenous identity as recorded on the Registry of Births Deaths and Marriages (RBDM)	Births in NSW 2001–2005	• The mother’s Indigenous status was highly consistent between the MDC and the RBDM
		An Aboriginal Statistical Variable (ASV) was created using the Indigenous identification in both datasets.	• The sensitivity was low in both data collections. At least one third of Indigenous mothers were not identified in the MDC and one-seventh in the RBDM
		The ASV was assessed by comparing numbers and percentages of births to Aboriginal mothers with the estimates by capture-recapture analysis	• Indigenous babies were more likely to be unregistered
Randall 2012 NSW	• Study examining mortality in Aboriginal and non-Aboriginal people after acute myocardial infarction in NSW	Used Aboriginal identifier based on the most recent public hospital admission for most of the analysis given recent improvements in Indigenous identification (88%), but undertook a sensitivity analysis based upon ‘ever identified’ and ‘all admissions’	• ‘Most recent’ identified 1183 (2.0%) of patients as Aboriginal; ‘ever identified’ identified 1479 (2.5%) and ‘all admissions’ identified 631 (1.1%) AMI patients as Aboriginal
			• In the fully-adjusted individual-level models, the ‘ever identified’ definition produced similar results to the ‘most recent’ definition, but the ‘all admissions’ definition resulted in higher odds of both 30-day and 365-day mortality for Aboriginal compared with non-Aboriginal patients

*For all studies linkage was based on probabilistic linkage. In WA this was undertaken by the WA Data Linkage Service and in NSW studies by the Centre for Health Record Linkage in NSW.

### Evaluation using self-reported Indigenous status

Bradshaw and colleagues followed-up of a cohort of 993 self-identified Aboriginal people who had participated in a cardiovascular risk assessment study, the Perth Aboriginal Atherosclerosis Risk Study (PAARS) in 1998/99 [23]. All participants had been initially recruited in the Perth metropolitan area through health, educational, public service and community institutions, and by family and community contact. The follow-up study used linkage of PAARS assessment data through WADLS to hospital separation (including renal dialysis) and death data to examine admissions and Indigenous status coding from 1980 to 2006. As non-Aboriginal people were not interviewed in this study, false positives and true negatives proportions could not be calculated.

The analysis found 14,413 admissions of PAARS participants, with only 39.9% of participants consistently recognised as Indigenous on every admission and 10% recognised as Indigenous on <10% of admissions. Admissions increased over time (as the cohort aged), with sensitivity of coding of Indigenous status improving from 0.8 in the early 1980s and exceeding 0.9 every year from 2002. Males were less consistently coded as Indigenous than were females. The authors concluded that Indigenous identification has improved sufficiently since 2000 for comparative studies of illness resulting in hospital admission in WA among Aboriginal and non-Aboriginal adults to now be undertaken with confidence [23].

### Studies of the impact of different identification algorithms on numbers and disease rates

Mak and Watkins linked data from mortality, hospitalisation, midwife registry and mental health datasets, incorporating census-derived population denominators, in order to improve the accuracy of Indigenous and non-Indigenous identity delineation in estimated notification rates for sexually transmitted infections (STIs) and blood borne viruses (BBVs) in WA [24]. STI and BBV notification rates according to Indigenous status in the original notification dataset were then compared with the improved data.

A quarter of the STI/BBV notifications received in 2004 had Indigenous status missing, with an identifier based on linkages available for 74% of the 1,959 notifications with missing Indigenous status. Rates of disease were then calculated, based upon different methods: the original identifier; “proportional assignment” whereby cases with an unknown identifier were distributed proportionally to the counts in Indigenous and non-Indigenous notifications; and ‘sensitive’ and ‘specific’ definitions of Indigenous identification from linked data (respectively: Indigenous identification in at least one record or consistently throughout the linked records). Following data linkage, the estimated rate of STIs and BBVs in the Indigenous population was lower, and the age-adjusted Indigenous:non-Indigenous rate ratios for both chlamydia and syphilis were significantly lower than previous estimates. The authors observed that Indigenous status influenced the likelihood of a missing Indigenous identifier, such that incompleteness of Indigenous status in STI/BBV data resulted in overestimation of the risk associated with Aboriginality for these diseases [24].

The identification of a substantial and steadily increasing proportion of deaths for which Indigenous status was not recorded in the WA state mortality database from 1997 (0.6% missing) to 2002 (6.6%) gave the impetus for a data linkage study undertaken by Draper and colleagues [25]. The mortality database was linked to the hospital morbidity, mental health and midwives databases. Two algorithms were developed, a conservative approach whereby status in matched data needed to be recorded as Indigenous in the majority of instances for an individual with missing status in mortality data to be considered Indigenous, and a more inclusive approach which classified a person as Indigenous if they had ever been identified as Indigenous in any of the linked databases. Life expectancy and all-cause mortality were then calculated, based upon the original identifier as well as the conservative and inclusive new Indigenous identifiers.

Data linkage enabled 95% of 1,784 deaths with previously unidentified Indigenous status to be allocated using the conservative approach (5.9% categorised as Indigenous) and 96% were allocated using the inclusive approach (7.5% categorised as Indigenous). Indigenous all-cause mortality for both sexes was higher than using the original identifier [25]. Both enhanced identifiers had an impact on estimated life expectancy, reflecting smaller improvements in life expectancy across the time period compared to the analysis using the original identifier. Thus, although Indigenous mortality rates had been falling, the under-ascertainment of Indigenous identity presented an overly optimistic picture of health improvement.

Briffa and colleagues investigated Indigenous status through record linkage of patients with cardiovascular disease (CVD) in hospital morbidity and mortality data [26]. The subjects were all those in WA with one or more public hospital CVD admissions during 2000–2005, and their index admission data were linked to 20-year admission history or a subsequent death record. Four approaches to Indigenous identification were used: using the identifier on (1) the *index* admission (baseline comparator); (2) the *index admission or subsequent death record*; (3) the *majority of records* (≥ 50% of hospital records) or in a subsequent death record; (4) *any Indigenous identification* in the hospital or death record (least conservative approach). The analysis examined how Indigenous identifiers were influenced by sex, age and region.

A total of 3,060 cases were identified as Indigenous in the original dataset, but this increased by 2.7% with the addition of the identifier from death records. The increment increased further to 3.7% for method (3) and to 20.8% based on an Indigenous identifier on any previous record. The results corresponded with underestimations of Aboriginal status in unlinked index admission data of 2.6%, 3.5% and 17.2% using the respective methods. The 8.5% of deaths for which Indigenous status was missing in death records and recorded as Indigenous in hospital morbidity data was about double the percentage of missing identifiers in the death records overall, indicating that misclassification is biased towards under-recording a person as Indigenous [26].

In a study examining Indigenous disparities in incident myocardial infarction and case fatality, Katzenellenbogen and colleagues analysed a person-linked file of admissions for myocardial infarction to any WA hospital for the period 1985–2004 and any matching death record using hospital morbidity and mortality datasets [27]. The WADLS was used to account for under- identification of Indigenous status, counting as Indigenous any person who had ever been identified as such on any hospital admission since 1980 or on their death record (inclusive definition). A sensitivity analysis was undertaken using a more restricted definition - those identifying as Indigenous on either their index admission or their death record. Age-standardised rates for total incidence were 10% lower in the 25–54 year age group and 14-18% in the 55–74 year age group using the restricted compared to the inclusive definition, with an inconsequential impact on non-Indigenous age standardised rates. Indigenous case fatality increased when the restricted definition was used [27].

A NSW paper by Neville and colleagues explored the impact of different ways of using the NSW Admitted Patients Data Collection (APDC) and mortality data to improve Indigenous identification by exploring the use of six different algorithms. Indigenous identification in death data alone was used as the baseline algorithm for comparison with five other combined-database algorithms with a spectrum of sensitivities for enhancing Indigenous identification (Table 2). Three of these algorithms included consideration of within- and between-facility consistency of Indigenous identification as well as single event identifiers [28]. Data linkage was useful for substantially improving the reporting of Indigenous deaths. The algorithm considered to be the most useful was based on the number of both APDC records and facilities reporting a person as Indigenous, providing 22.7% enhancement of Indigenous identification for deaths overall, with a marked progressive reduction in misclassified status in death records of Indigenous people in relation to remoteness (44.5% in major cities compared to 24% for regional areas and 4.5% in very remote areas).

A multilevel data linkage study by Randall et al. (2012) examined mortality risk following myocardial infarction in Aboriginal and non-Aboriginal people in NSW [29]. They utilised a definition of Indigenous status based upon the person’s most recent admission, but undertook a sensitivity analysis based on two alternative classifications for Aboriginal status, ‘ever identified’ and identified in ‘all admissions’. The different identifiers resulted in very different numbers of patients classified as Aboriginal (‘most recent’ 1183 [2.0%]; ‘ever identified’ 1479 [2.5%]; ‘all admissions’ 631 [1.1%]). In the fully adjusted individual-level models, the ‘ever identified’ and ‘most recent’ definitions produced similar mortality outcome estimates, but the ‘all admissions’ definition resulted in a higher odds ratio for both 30-day and 365-day mortality for Aboriginal compared to non-Aboriginal people.

### Cross referencing of databases and capture-recapture

Xu *et al.* used linked records (2001–2005) from the NSW Midwives Data Collection (MDC) and the Registry of Births Deaths and Marriages (RBDM) in a predominantly methodological paper to demonstrate substantial underestimation in both databases of the number of newborns with mothers identifying as Indigenous [30]. Acknowledging the absence of a ‘gold standard’ measure for comparison, the authors calculated the consistency in Indigenous identification between the two databases, as well as the sensitivity and specificity of each with respect to the other. The sensitivities of the respective databases were 67% and 86%. Unsurprisingly, given the overwhelming proportion of individuals identified as non-Indigenous in both databases, the consistency and specificity of Indigenous identification were very high (≥99%). Additionally, they calculated an ‘Aboriginal statistical variable’ (ASV), classifying as Indigenous any newborn whose mother self-reported as Indigenous in either record and ‘missing’ if Indigenous status was unrecorded in both databases, with the remainder classified as ‘non-Indigenous’ Based on this measure, the proportion of mothers in the MDC identified as Indigenous increased from 2.6% to 3.3%, and in the RBDM from 2.3% to 2.6%. Further, capture-recapture analysis based on the two databases was used to estimate the total number of newborns with mothers identifying as Indigenous. The number identified (17,312; 3.9% of total population) was markedly higher than the 11,349 and 9,181 identifiable in the respective databases without data linkage, suggesting that even when using identifiers from both data sets, the number of Indigenous births are under-estimated. The authors did not interrogate or discuss the complexity introduced by the varying criteria for attribution of identity between the two datasets - in the MDC, a newborn’s Indigenous identity is that recorded for the mother, whereas the RBDM records the Indigenous identification of both parents. The authors did not apply the findings of this study to any particular health outcomes.

There were no papers identified from any other jurisdictions on data linkage and ascertainment of Indigenous status.

## Discussion

The issue of identification of Indigenous and minority populations is not unique to Australia. The quality of the long-established Western Australian data linkage system allows insights not necessarily available elsewhere. The National Collaborative Research Infrastructure Strategy (NCRIS), an initiative of the Australian Government to develop research infrastructure in selected priority areas for science, technology and the health and wellbeing of the Australian population, in 2006 allocated $20 million over three years to further develop Australia’s population health and clinical data linkage capability. As a result of this, data linkage capacity is being developed in other states of Australia. The Centre for Health Record Linkage (CHeReL) in NSW currently enables linkage only of more recent administrative data but is rapidly increasing the number of linkages, fostering research, population health surveillance and health system performance monitoring, exemplified by the three NSW papers cited above [31]. Other states are also developing their capacity. This systematic appraisal brings together the peer-reviewed Australian work to date on efforts to evaluate and improve the ascertainment of Indigenous status in administrative data collections through data linkage. The included studies all assessed the impact of different approaches to Indigenous identification on health outcome estimates and highlight the usefulness of data linkage for exploring the impact of minority under-identification in routinely-collected health data. These approaches can also provide insight into changes in Indigenous identification over time.

While there is a standard definition of an Aboriginal and Torres Strait Islander, how this is ascertained differs based upon the circumstances in which the data are collected. Within hospitals, a ward or emergency department clerk generally collects demographic information; and at least some people are reluctant to ask questions on Indigenous status, out of concern for causing offense [15]. Training in how to ask the question is important, and differences in the way the question is asked or perceived differences in how Indigenous people are treated may contribute to some of the variability in an individual’s identification. There has been increased attention in recent years to training of staff about how to ask people their Indigenous status. Indigenous status in mortality data is based on information provided by funeral directors, who rely upon a relative to provide information. Adjustment of the mortality identifier also may occur within the ABS unit (see Figure 1). Although infectious disease notification data are provided by both laboratories and diagnosing doctors, only clinicians have the requisite contact with the patients to ask them whether they are Indigenous. The Indigenous status field in medical records is notoriously under-completed, and errors of under-ascertainment from an initial event are most likely replicated after the re-completion of the demographic data during subsequent contacts. Under-ascertainment of Indigenous status in general practice is well documented, with limited recent improvement despite government efforts and incentives [32].

Under-ascertainment of Indigenous status generally reduces estimates of the magnitude of Indigenous disadvantage, with more inclusive indigenous identifiers apparently increasing health differentials as shown by Draper and colleagues for mortality rates/life expectancy [25] and by Katzenellenbogen et al. for myocardial infarction incidence [27]. In the NSW paper by Randall, the more restrictive definition of Indigenous status (“all admissions”) led to a significant increase in the relative odds of both 30-day and 365-day mortality following an admission with acute myocardial infarction [29]. However, Mak and Watkins [24] found that disease rates and Indigenous:non-Indigenous rate ratios decreased with improved identification, suggesting a substantial bias towards the under-recording of non-Indigenous status in STI/BBV data, diseases that are often considered to be socially stigmatised. A similar phenomenon has been reported previously by Condon in relation to doctor notification of gonorrhoea in WA where infections in Indigenous people were more likely to be notified than cases in non-Aboriginal people [33].

Draper and colleagues had noted deterioration in the recording of Indigenous status in mortality data and attributed this to diminishing efforts to follow up missing data. Briffa et al’s linkage of hospital and death records also suggests that Aboriginal status is under-identified in official death records by about 27% in WA, similar to the 26.3% reported by Neville et al. in NSW using the algorithm they considered most promising [26]. Under-identification of Indigenous people occurs more in urban areas where the proportion of the population that is Indigenous is lowest [15,26], in older people [26,27], and in those who are least disadvantaged [26]. Similar findings with regard to demographic factors associated with under-identification were reported by Randall and colleagues from NSW [29].

Bradshaw and colleagues’ approach of linking administrative data to an identified cohort noted that Indigenous ascertainment in hospital data has been improving with time [23], although individuals in the cohort all self-identified as Indigenous at a time when there was a resurgence of Indigenous identification. To place this in historical context, recruitment was occurring within a couple of years of the publication in 1997 of the “Bringing them Home” report which documented for the first time the policies and impact of the ‘stolen generations’, during which children of Indigenous Australians were systematically removed from their families by government agencies [34]. Publicity regarding the National Inquiry into the forced removal and separation of Aboriginal children from their families upon which this report was based is likely to have given Aboriginal descendants of families affected by forced removal validation of their heritage and the courage and legitimacy to identify as Indigenous.

As shown in this paper, the use of linked data to interrogate Indigenous identification and its impact on Indigenous health outcomes has been relatively uncoordinated, being led by different analytic groups around Australia - in particular WA and NSW - who have developed approaches of varying complexity and employing different data sources. In mid-2012, at the time of finalising this manuscript, a federal government publication was released that outlined national best practice guidelines for data linkage activities related to Indigenous Australians [35]. These guidelines provide a framework in which data linkage occurs in this context, and outline options that can be used to derive more accurate Indigenous data at both individual unit record and aggregate levels. The guidelines are not prescriptive and draw on the growing experience in Australia as reflected in this paper. In addition, linking of data across administrative datasets to improve the estimation of Indigenous status is beginning to occur in reporting of administrative data [36] and increasing refinement of approaches is likely to occur in the coming years.

Methods of ascertainment of Indigenous status in administrative data in Australia differ from those in some other countries, in which it may be based upon an identity card, blood rule or require ties to a reservation. Ross has pointed out that ‘ethnic identity is fluid and capable of change over the course of a lifetime and within specific situations’ [37], and quotes Waters that ‘multiple ancestries exist among a large proportion of the population (…) [but] people often choose or are forced into one category for purposes of administrative classification or counting schemes’ [38] (p3). As well as these issues of identity, there are other reasons given for Indigenous people for disclosing or not their Indigenous identity including the way the question is asked, whether any negative consequence or advantage arises from disclosure. The question may have not been asked because of assumptions made by the person completing the information or the circumstances of the situation such as in an emergency situation, and it may be provided differently in when there is the opportunity to self-enumerate compared to when it is collected by a third party, such as a hospital clerk or a funeral director or provided by a parent or other relative on behalf of a child. Given this fluidity of identification, data linkage has proved to be a useful tool for evaluating Indigenous identification in WA and in NSW. Rather than provide ‘correction factors’, papers are now able to report results using different identifiers, effectively undertaking sensitivity analyses under different identification scenarios. Until the validity of Indigenous identification improves consistently, there is value in Indigenous health studies providing sensitivity ranges in which the true parameters lie, thereby making explicit the inherent instability of Indigenous identification as well as the measurement error that is ubiquitous in administrative health data. In descriptive studies, that means providing different estimates of the number of cases and/or rates. Similarly, separate regression models can be run to evaluate whether measures of effect change when different Indigenous identification algorithms are used.

The substantial health differentials between Indigenous and non-Indigenous Australians only really came to public consciousness after a referendum in 1967 which allowed Indigenous Australians to be counted in the Census enabling them to be identifiable as a minority group. Improved identification in administrative health and census data is an important component of measuring disadvantage and therefore of assessing progress in reducing health disparities. In the long term, it can contribute to efforts to change the reality of poor quality and quantity of life for Indigenous people and to alter policy initiatives to improve the health of Indigenous populations. Reliable data are required for accountability at political, policy and implementation levels, and the papers reviewed here contribute to efforts to monitor accurately the health of Indigenous people in Australia. These approaches also have applicability to other countries that have the potential to link health survey and administrative records.

## Conclusion

Data linkage can contribute to national efforts to improve the quality of Indigenous data. Among Australian jurisdictions, WA has the longest history of using linked data in epidemiological analysis, but NSW and other Australian states are now developing data linkage capability. It will be possible to utilise the approaches reviewed in this paper to enhance the accuracy of reporting of Indigenous health outcomes. The challenges of Indigenous identifiers are changing with and over time, and population denominators (based on Census data) have also changed with respect to increasing Indigenous identification [39]. The calculation of rates and trends in health status without a stable means of identifying Indigenous Australians in both administrative and Census data is fraught with challenges and seem likely to persist. Efforts to improve on data accuracy at the point of data collection remain important in improving the quality of Indigenous health data.

## Competing interests

The authors declare that they have no competing interests.

## Authors’ contributions

ST and JK conceived the study and undertook the initial literature search. JW repeated the literature search and undertook further retrieval of publications. ST and JK reviewed the retrieved publications for eligibility for inclusion in the study. All authors participated in writing the manuscript and approved the final draft.

## Pre-publication history

The pre-publication history for this paper can be accessed here:

http://www.biomedcentral.com/1472-6947/12/133/prepub
